# Accuracy of motion quantification in dynamic CT images of the wrist joint across multiple CT vendors: A phantom study

**DOI:** 10.1002/mp.70667

**Published:** 2026-09-03

**Authors:** Hanne Vries, Brigitte van der Heijden, Stefan Hummelink, Iwan Dobbe, Ioannis Sechopoulos, Geert Streekstra

**Affiliations:** ^1^ Department of Plastic and Reconstructive Surgery Radboud University Medical Center Nijmegen the Netherlands; ^2^ Department of Medical Imaging Radboud University Medical Center Nijmegen the Netherlands; ^3^ Amsterdam UMC location, Biomedical Engineering and Physics University of Amsterdam Amsterdam the Netherlands; ^4^ Department of Plastic and Reconstructive Surgery Jeroen Bosch Hospital ’s‐Hertogenbosch the Netherlands; ^5^ Technical Medicine Center University of Twente Enschede the Netherlands; ^6^ Dutch Expert Center for Screening (LRCB) Nijmegen the Netherlands; ^7^ Musculoskeletal Health ‐ Restoration and Development Amsterdam Movement Sciences Amsterdam the Netherlands

**Keywords:** 4D CT, image registration, motion artifacts

## Abstract

**Background:**

Four‐dimensional computed tomography (4D CT) allows dynamic assessment of wrist kinematics and offers a non‐invasive alternative for evaluating ligament instability. However, insufficient temporal resolution and differences in acquisition or reconstruction protocols may introduce motion‐related artifacts that affect quantitative analysis. As these artifacts can mimic pathological carpal motion, measurement error must be quantified to distinguish true pathology from methodological error. Therefore, systematic evaluation of motion quantification across different CT systems and acquisition protocols is required.

**Purpose:**

To evaluate the errors in motion quantification across acquisition and reconstruction protocols of different CT systems in 4D CT imaging of wrist bones.

**Methods:**

A rotating wrist phantom with three 3D‐printed bones (scaphoid, lunate, capitate) was scanned on five CT systems from three manufacturers (Aquilion ONE PRISM and ONE Vision, Canon Medical; Revolution Apex, GE HealthCare; single‐source and dual‐source SOMATOM Force, Siemens Healthcare). One static 3D scan and multiple 4D scans were acquired at different phantom rotation speeds, each lasting 10 s. Single‐source covered 0.050–0.300 phantom rotations per second (rps), and dual‐source 0.100–0.600 rps. Dose dependence was evaluated on two systems (80 kV/40 mA and 120 kV/100 mA). Images were reconstructed in full and partial modes, segmented, and registered using point‐to‐image registration. Motion quantification error was calculated for translation and rotation of the scaphoid and capitate relative to the lunate, referenced to the static scan, and reported per rotation cycle. Motion quantification error was additionally expressed as a function of normalized motion per reconstructed frame to relate the error to the amount of motion occurring during image acquisition. Effects of CT system, bone type, reconstruction method, and rotation speed were analyzed using a linear mixed model. Statistical significance was defined as *p* < 0.05.

**Results:**

The motion quantification error was hardly affected by dose. For 0.100 rps, the errors of the best single‐source system were median 0.29 mm (interquartile range: 0.21–0.36 mm) and 1.37° (0.82°–2.18°) for full and 0.21 mm (0.16–0.25 mm) and 0.70° (0.52°–0.95°) for partial reconstructions for the capitate; the dual‐source system showed the lowest errors (0.14 mm (0.12–0.17 mm) and 0.48° (0.39°–0.62°)). Motion quantification error scaled approximately linearly with normalized motion per reconstructed frame, corresponding to approximately 15% of the motion occurring during one reconstructed frame.

**Conclusions:**

Motion quantification errors were largely determined by phantom rotation speed and temporal resolution. The reported values provide a basis for defining the detection threshold for quantitative wrist kinematics, supporting differentiation between true kinematics and apparent displacement caused by motion artifacts.

## 1 INTRODUCTION

Wrist injuries, such as ligament ruptures, require early and accurate diagnosis. Delayed or missed diagnosis can lead to instability and subsequently to the development of osteoarthritis, a degenerative joint disease causing chronic pain, reduced range of motion, and functional impairment.^1^ The evaluation of wrist ligament injuries is currently based on clinical findings, arthroscopy, and static imaging techniques such as radiography, Computed Tomography (CT), and Magnetic Resonance Imaging (MRI).^2^, ^3^, ^4^ MRI arthrography is widely used for direct visualization of intrinsic wrist ligaments due to its high soft‐tissue contrast and ability to show ligament morphology. However, like conventional CT and MRI, it remains a static assessment. Moreover, ligament visualization alone does not provide information on whether a lesion is associated with abnormal carpal kinematics, which underlies wrist instability and symptoms.^5^

In selected cases, fluoroscopy is performed to visualize wrist motion.^6^ However, this technique suffers from superimposition of the carpal bones, and often requires the radiologist to perform passive wrist movement, which may not represent physiological motion. Dynamic MRI is emerging as a promising radiation‐free technique for assessing wrist kinematics and has shown potential for diagnosing partial and complete scapholunate (SL) lesions. Nevertheless, current dynamic MRI techniques still require a trade‐off between temporal and spatial resolution, limiting their clinical performance.^2^, ^7^, ^8^

Four‐dimensional computed tomography (4D CT) offers a non‐invasive, dynamic alternative that combines high spatial and temporal resolution, allowing assessment of wrist motion during active movement of the hand.^9^, ^10^, ^11^, ^12^, ^13^ This enables the carpal bones to be captured in 3D throughout the motion cycle, providing both qualitative and quantitative assessment of bone translations and rotations. However, the large amount of data generated by 4D CT makes manual interpretation by radiologists too time consuming. Consequently, automated analysis methods are preferred to objectively quantify wrist motion. By extracting kinematic parameters such as intercarpal distance and rotation angles, a quantitative assessment of wrist motion can be performed to identify abnormal motion patterns that may indicate a wrist injury.^14^, ^15^, ^16^, ^17^

For clinical application, the ultimate goal of quantitative 4D CT is to establish diagnostic thresholds that distinguish physiological from pathological wrist motion.^18^ However, before diagnostic thresholds can be meaningfully defined, the detection threshold of the automated analysis must first be established, defined as the smallest kinematic difference that can be confidently attributed to true bone motion rather than methodological error. Without this, small observed differences in inter‐bone motion may be interpreted as pathological instability, whereas they may simply reflect methodological variation within the imaging pipeline.

Accurate segmentation and registration of the carpal bones are required for such automated kinematic analysis, and their performance depends strongly on the quality of the acquired scans. In dynamic imaging, temporal resolution plays an important role in the quality of motion capture. If the temporal resolution is insufficient relative to the speed of the wrist motion, the resulting scans may exhibit motion‐related artifacts such as blurring or ghosting.^19^, ^20^ These artifacts can introduce errors in the segmentation and registration, affecting the derived kinematic parameters.^21^ As shown by Dobbe et al., variations in acquisition parameters such as radiation dose have a minimal influence on registration accuracy, while motion artifacts do have a major impact on the accuracy of the kinematic analysis.^22^ Reducing wrist speed to minimize motion artifacts may improve image quality but can limit how accurate the captured motion represents natural wrist movement during daily activities, potentially diminishing the clinical value of a 4D CT scan.

Image quality can also vary due to differences in CT system hardware, and in acquisition and reconstruction protocols.^9^ Commonly used scanners allow reconstructions from partial gantry rotations, reducing acquisition time and thereby limiting motion artifacts. In addition, dual‐source CT (DSCT) systems can further improve temporal resolution.

Although several studies have demonstrated the feasibility of dynamic wrist imaging using single‐source CT (SSCT) systems, systematic comparisons across different CT scanners and acquisition or reconstruction protocols remain limited.^12^, ^23^, ^24^, ^25^, ^26^ Therefore, the aim of this study is to evaluate errors in motion quantification across acquisition and reconstruction protocols of different CT systems in 4D CT images of wrist bones.

## 2 METHODS

In this study, a rotating phantom representing three wrist bones was used to evaluate the effect of motion artifacts on motion quantification accuracy across several CT systems, including both single‐ and dual‐source acquisitions. Throughout the manuscript, a spiral 3D scan is defined as a single spiral 3D CT scan during which the phantom remained static. A 4D CT scan is defined as a sequence of axial 3D CT scans over time, during which the phantom could be either static or in motion, depending on the experiment conducted, as described below and listed in Table 1. After acquisition, the images were reconstructed using various protocols and analyzed to quantify the methodological errors introduced by the complete 4D CT imaging and analysis pipeline, including image acquisition, reconstruction, segmentation, and registration. These errors, resulting from reconstruction artifacts, dose‐related effects, and/or motion artifacts, are hereafter referred to as motion quantification error.

**TABLE 1:** Acquisition and reconstruction parameters of the five different CT systems.

Parameter	WACT‐P		WACT‐V		WACT‐R		SSCT		DSCT	
Manufacturer	Canon		Canon		GE		Siemens		Siemens	
CT system	Aquilion ONE PRISM		Aquilion ONE Vision		Revolution Apex		SOMATOM Force		SOMATOM Force	
Scanning mode	Spiral 3D	Axial 4D	Spiral 3D	Axial 4D	Spiral 3D	Axial 4D	Spiral 3D	Axial 4D	Spiral 3D	Axial 4D
Tube voltage [kV]	120	80	120	80	120	80	120	120	120	80
Tube current [mA] or Tube current–time product [mAs]	100	40	100	40	50	40	70	30	70	30
Rotation time [ms]	275	275	350	350	280	280	250	250	250	250
Helical scan length [cm]	10		12		10		8.58		9.78	
^a^Total collimation [no. rows x detector width in mm]	–	160 × 0.5	–	160 × 0.5	–	128 × 0.625		96 × 0.6		96 × 0.6
Acquisition duration [s]	3	10.20	3	10.45	3	10.10	3.75	10.10	3.75	1.65
Temporal resolution [ms]										
Full recon	275			350		280		250		66
Partial recon	175			213				181		
Field of view [mm]	150	150.40	150X	150	154	154	154	154	154	160
Pixel size [mm]	0.29	0.29	0.29	0.29	0.30	0.30	0.30	0.30	0.30	0.31
Slice thickness [mm]	0.5	0.5	0.5	0.5	0.6	0.6	0.6	0.6	0.6	0.6
Slice increment [mm]	0.3	0.3	0.3	0.3	0.3	0.6	0.3	0.3	0.3	0.3
Frame interval [ms]	–	300	–	303	–	280	–	303	–	133
Reconstruction kernel	BONE	BONE	FC30	FC30	BONE PLUS	BONE PLUS	Br64s∖2	Br64d	Br64s∖2	Br64d∖3
Reconstruction technique	AiCE	AiCE	AIDR 3D	AIDR 3D	AR20	AR20	ADMIRE	ADMIRE	ADMIRE	ADMIRE
^b^CTDI_vol_ [mGy]	4.40	4.80	7.30	10.90	6.85	8.28	4.70	65.89	4.35	2.90

Abbreviations: DS = dual source; SS = single source; WA = wide angle.

^a^ The WACT‐P and WACT‐V systems provide a maximum detector coverage of 320 × 0.5 mm (160 mm); however, an 80 mm detector configuration (160 × 0.5 mm) was used in this study.

^b^ CTDI_vol_ values are based on measurements in the standard 32‐cm CTDI body phantom.

### 2.1 Phantom

**FIGURE 1:**
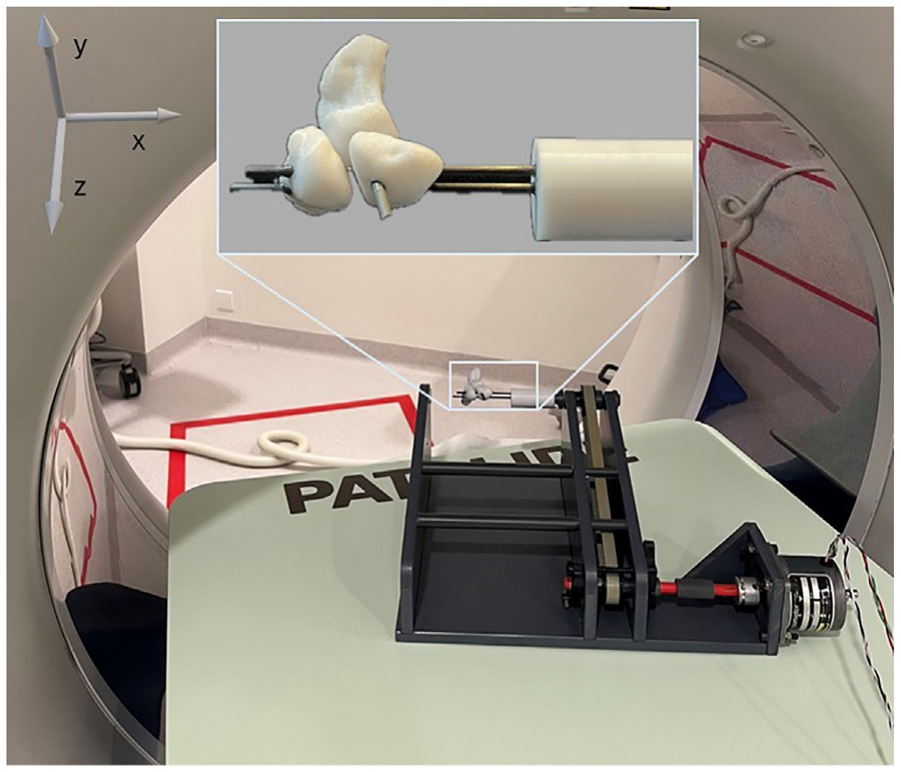
Phantom setup for scan acquisition. The phantom was oriented such that the angle between the rotational axis of the phantom and the *x* axis is approximately 15°, to avoid excessive x‐ray attenuation.

To simulate the motion of carpal bones during 4D CT scanning, we used a phantom that consists of three 3D‐printed bones (Polycarbonate ISO 10993), representing the scaphoid, lunate, and capitate. The three bones were mounted in anatomical positions relative to each other using carbon rods, with the centroid of the lunate placed at the axis of rotation (Figure 1). This placement is expected to minimize motion artifacts at the lunate and increase them distally in the capitate, consistent with artifact patterns typically observed in dynamic wrist imaging.^19^ A programmable stepper motor (Model C57‐51, CV series; Parker Hannifin Corp., Rohnert Park, California, USA) was used to rotate the phantom, allowing for user‐selectable and known angular velocity of the 3D‐printed carpal bones.

### 2.2 Image acquisition

The spiral 3D CT scans and axial 4D CT scans were acquired with five different CT systems from three CT manufacturers, one in two different acquisition modes: Wide angle CT‐P (WACT‐P): Aquilion ONE PRISM (Canon Medical Systems, Otawara, Japan); Wide angle CT‐V (WACT‐V): Aquilion ONE Vision (Canon Medical Systems); Wide angle CT‐R (WACT‐R): Revolution Apex (GE HealthCare, Chicago, USA); SSCT and DSCT: SOMATOM Force (Siemens Healthcare, Forchheim, Germany). For the purposes of this study, from here on the single‐source and dual‐source acquisition modes of the same scanner (i.e., Siemens SOMATOM Force) are denoted as separate CT systems, allowing their performance to be evaluated and communicated independently.

For all scans, the phantom was placed at the isocenter of the scanner, with the rotational axis of the phantom parallel to the *x*‐*z* plane (Figure 1). To avoid excessive and unrealistic x‐ray attenuation by the platform holding the phantom, the angle of the rotational axis with the *x* axis was approximately 15°.

#### 2.2.1 Single‐source systems

For the single‐source acquisitions (WACT‐P, WACT‐V, WACT‐R, SSCT), 4D CT scans were obtained at seven different phantom rotation speeds; 0.050, 0.075, 0.100, 0.125, 0.150, 0.200, and 0.300 rotations per second, all with an acquisition time of 10 s each. These phantom rotation speeds were chosen to encompass the range of temporal rates of wrist motion cycles seen clinically. For example, a typical clinical flexion‐extension scan involves one motion cycle of a complete flexion–extension–flexion movement, resulting in wrist moving approximately 260° degrees in 10 s. This is comparable to the 0.075 rotations per second of the phantom.

#### 2.2.2 Dual‐source system

Acquisition of the dual‐source scans was only possible with the DSCT scanner using an electrocardiogram‐gated cardiac CT protocol. Therefore, this protocol was simulated with an electrocardiogram pulse at 30 beats per minute, so that the acquisition time of one 4D CT image sequence was 2 s. With a time increment between the motion phases of 10%, this resulted in 15 volumes acquired per 4D CT sequence. For DSCT, the phantom rotations speeds were set to 0.100, 0.200, 0.300, 0.400, 0.500, and 0.600 rotations per second.

Details of the 3D and 4D scanning protocols for the different CT‐scanners are listed in Table 1.

### 2.3 Image analysis

The image analysis method quantifies the apparent motion between the capitate or scaphoid and the lunate in the 4D CT scans relative to their positions in the 3D scan. To this end the bones were segmented in the spiral CT scan and subsequently registered to all time frames of the 4D CT scans, as proposed and evaluated by Dobbe et al.^22^ Briefly, segmentation was based on region‐growing, which initialized a level‐set segmentation algorithm, and subsequent mesh extraction using the marching cubes algorithm. The resulting segmentation was visually verified before proceeding. Following segmentation, for each bone, a double‐contour polygon was extracted and gray levels subsampled from the spiral 3D scan were assigned to the mesh points. This enabled rigid registration of each mesh to the time frames of the 4D CT scans. For the first frame, a manual initialization was used to place the contour on the target bone and provide a starting position for the registration algorithm, after which rigid registration automatically refined the alignment. Successful alignment was subsequently confirmed using the registration metric. The reproducibility of the segmentation and manual initialization was assessed by repeating the segmentation and first‐frame initialization five times for a representative dataset (WACT‐P).

Registration was based on the Nelder–Mead downhill simplex optimization algorithm^27^ with a six‐parameter search space, providing the transformation matrices for the lunate, scaphoid, and capitate (**M** *_L_*, **M** *_S_*, **M** *_C_*, respectively), from which three translations (Δ*x*, Δ*y*, Δ*z*) and three rotations (Δϕ_x_, Δϕ_y_, Δϕ_z_) could be obtained. The position of the bone meshes obtained from the spiral 3D scan served as the reference. The lunate was selected as the reference bone, as the functional rotation axis of the wrist passes close to its centroid. The relative motion of each bone *B* (scaphoid or capitate) with respect to the lunate in time frame i was expressed as TM_relative_ = TM_L_^−^^1^ · TM_B_, which describes each bone's position in the lunate's coordinate frame and cancels out any global wrist motion. As the analysis is based on relative transformations, no additional anatomical local coordinate system was required. The apparent motion in each time frame *i* was then expressed by the total translation error defined as $\Delta t_{\mathrm{total}}=\sqrt{\Delta_{x}^{2}+\Delta_{y}^{2}+\Delta_{z}^{2}}$ and the total angular error defined as $\Delta \varphi_{\mathrm{total}}=\sqrt{\Delta \varphi_{x}^{2}+\Delta \varphi_{y}^{2}+\Delta \varphi_{z}^{2}}$.

### 2.4 Experiments

To determine what the effects of reconstruction method, dose, and temporal resolution were on the motion quantification accuracy, three different experiments were conducted. All acquisitions were performed using the clinical imaging protocol for wrist ligament injuries (Table 1), except where stated otherwise.

#### 2.4.1 Motion quantification error for full and partial reconstructions in a static phantom

To evaluate whether the CT acquisition, reconstruction, and analysis introduce apparent bone displacement in the absence of motion, a spiral 3D CT scan and an axial 4D CT scan were acquired while the phantom remained stationary with all five CT systems. After acquisition, the 4D CT scan was reconstructed using full and partial reconstructions, as partial reconstructions may be more prone to reconstruction artifacts.^22^ Partial reconstructions were not available on this GE CT system. Therefore, only full reconstructions were evaluated for this scanner. The relative position of the capitate with respect to the lunate, as determined from the spiral 3D scan, was used as reference and compared with the corresponding relative position obtained after 4D CT acquisition and analysis. Since no actual motion occurred, this stationary experiment served as an independent assessment of segmentation and registration accuracy in the absence of motion. Therefore, any measured displacement reflects solely the variability introduced by the image‐analysis pipeline, rather than true movement.

#### 2.4.2 Motion quantification error versus radiation dose

Because the scanner comparisons were performed using the clinically implemented acquisition protocols of each system rather than dose‐matched protocols, additional experiments were conducted to assess the influence of radiation dose on motion quantification accuracy. These dose‐related effects were evaluated by acquiring 4D CT scans at multiple dose levels while the phantom was rotating. This was performed only for WACT‐P and DSCT, representing commonly used single and the dual source scanners. Since preliminary analysis indicated little dose‐dependent effect on motion quantification accuracy, no additional scanners were included. See Table S1 for the dose protocols for all phantom rotation speeds stated in the imaging acquisition section. The 4D CT scans were again reconstructed using both full and partial reconstructions.

#### 2.4.3 Motion quantification error versus temporal resolution

After accounting for the reconstruction and dose‐related effects, the motion quantification accuracy in the subsequent experiment was expected to primarily reflect motion‐induced artifacts. Therefore, the influence of temporal resolution on motion quantification accuracy was evaluated for all CT systems and phantom rotation speeds, using both full‐ and partial reconstruction where available. For systems without partial reconstruction functionality, only full reconstructions were analyzed. The results are reported as a function of temporal resolution, since this parameter varied substantially across scanners and reconstruction modes.

Moreover, to compare across scanners and reconstruction protocols, an additional normalized motion parameter was calculated, representing the amount of capitate motion occurring during acquisition of a single reconstructed frame. For translation errors, the normalized motion parameter was defined as the capitate displacement per frame (mm). This was calculated using 2π f∙T∙r, where f is the phantom rotational frequency (rps), T is the temporal resolution of the reconstructed 3D volume (s), and r is the radius of rotation defined by the capitate centroid distance to the phantom rotation axis (16.3 mm; Figure SC1). For rotational errors, the normalized motion parameter was defined as the angular displacement per frame (degree), calculated as 360° f∙T. The relationships between the normalized motion parameter and the errors in translation and rotation were evaluated using pooled linear regression across all scanner systems and reconstruction modes.

### 2.5 Statistical analysis

All translational and rotational errors were summarized using the median and 25^th^–75^th^ percentiles for each condition tested, which were used as input for statistical testing.

To evaluate the effects between the scanners, phantom rotation speeds and bone type on the error in motion quantification, two statistical models were created. The first was a linear mixed‐effects model focusing on the SSCT systems (WACT‐P, WACT‐V, WACT‐R, and SSCT). Bone type, reconstruction method, and phantom rotation speed were included as fixed effects, while CT system was included as a random effect to account for inter‐scanner variability. The second was a standard linear model evaluating partial reconstructions across WACT‐P, SSCT, and DSCT. Bone type, CT system, and phantom rotation speed were included as fixed effects, with CT system incorporated as a comparison factor to allow direct assessment of scanner‐specific differences. The second model evaluated partial reconstructions across WACT‐P, SSCT, and DSCT using bone type, CT system, and phantom rotation speed as fixed effects, with CT system included as a fixed comparison factor to allow direct assessment of scanner‐specific differences.

Effect sizes were reported as partial η^2^. Statistical significance was defined as *p* < 0.05. All statistical analyses were performed in RStudio (R Foundation for Statistical Computing, Vienna, Austria). See Supplementary A for the statistical analysis of the dose protocols.

## 3 RESULTS

**FIGURE 2:**
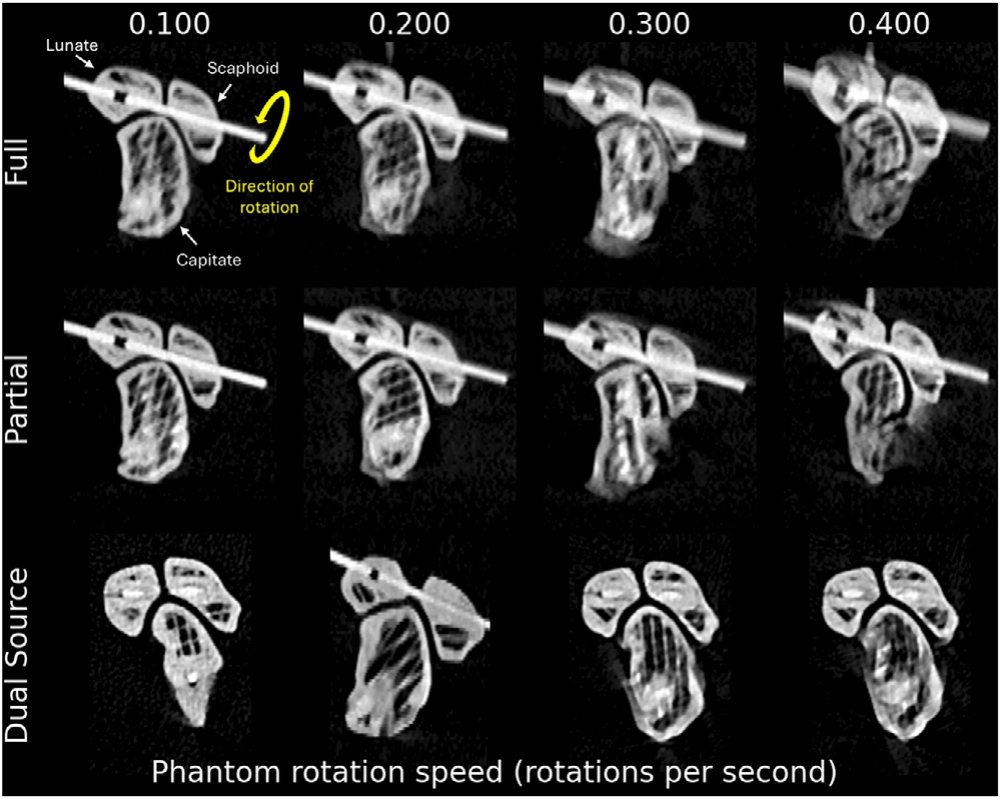
Example of 4D CT scans of the dynamic wrist phantom reconstructed with full, partial, and dual‐source methods at increasing phantom rotation speeds (0.100–0.400 rotations per second). In the single‐source system (WACT‐P), motion artifacts, shown as image blurring, become increasingly visible in the full reconstructions at speeds above 0.200 rotations per second, whereas partial reconstructions show fewer blurring artifacts. The dual‐source system (DSCT) results in substantially reduced motion artifacts across all speeds.

The effect of image acquisition and reconstruction methods on the CT image quality is visualized in Figure 2, where the full reconstructions in WACT‐P show increasing blurring and ghosting artifacts at higher phantom rotation speeds, while partial reconstructions result in less blurring and ghosting. Although the partial reconstructions appear with less blur, they also exhibit subtle image distortions that are not observed in the corresponding full reconstructions. In comparison, DSCT gives the most stable image quality with fewer motion artifacts across all speeds. Repeating the segmentation and manual initialization five times for a representative dataset resulted in a maximum difference in capitate position of 0.013 mm in translation and 0.062° in rotation. Visual inspection confirmed that all segmentations were correct, and no manual corrections were required.

### 3.1 Motion quantification error for full and partial reconstructions in a static phantom

**FIGURE 3:**
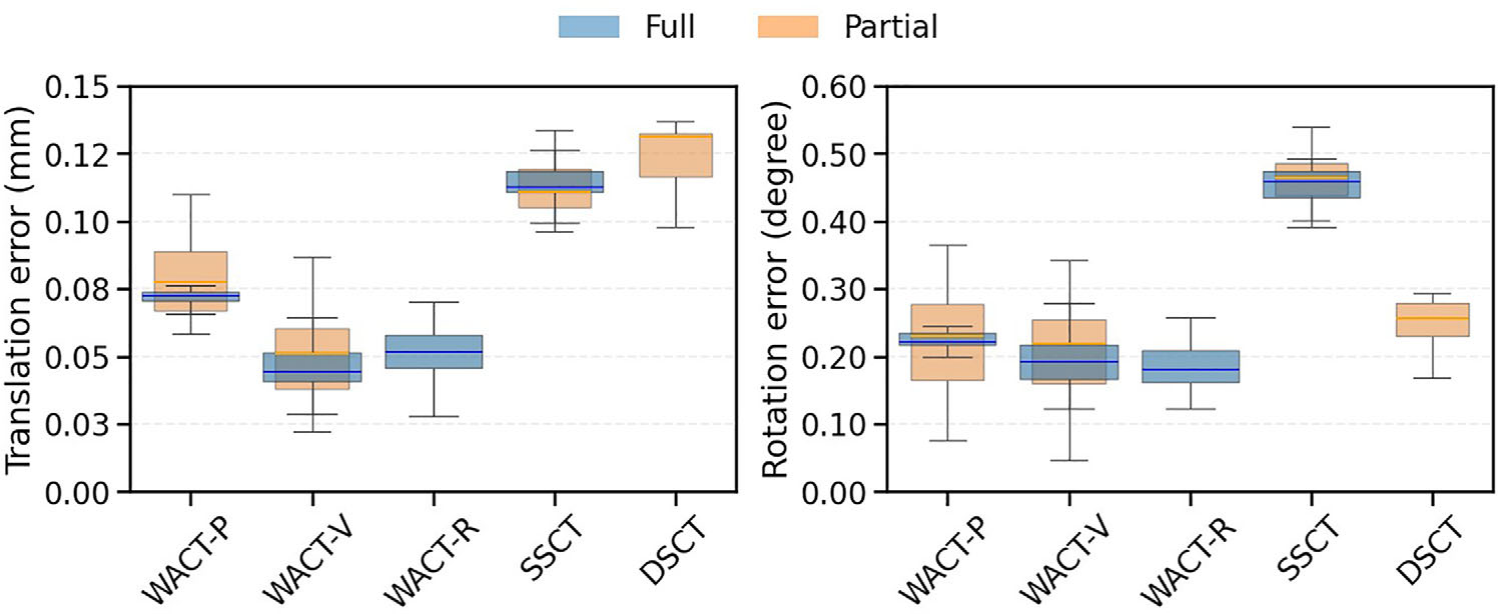
Motion quantification errors following 4D CT acquisition and analysis for the capitate relative to the lunate (reference) in a static phantom, using single‐source scanning (WACT‐P, WACT‐V, WACT‐R, SSCT) and dual source scanning (DSCT). Boxplots show the distributions for reconstructions from full rotation and partial rotation acquisitions (WACT‐P, WACT‐V, SSCT). The central line represents the median, the box indicates the interquartile range (Q1–Q3), and the whiskers extend to 1.5× the interquartile range.

In all three CT systems that allowed for partial‐rotation reconstructions, the measured capitate displacement in the static phantom did not differ between the full and partial reconstructions (Figure 3). The variability (interquartile range) in the translations and rotations was slightly larger for the partial reconstructions compared to that of the full reconstructions, remaining within 0.15 mm and 0.5° for all systems. This indicates that, although small non‐zero median errors were present, no consistent directional shift between full and partial reconstructions was observed. Therefore, both reconstruction types were included in the next experiments.

### 3.2 Motion quantification error versus radiation dose

Varying the radiation dose did not significantly affect motion quantification accuracy for either WACT‐P or DSCT (Figures SA1 and SA2). No significant differences in translation or rotation errors were observed between dose levels for either bone at any phantom rotation speed (all p > 0.76). Therefore, the lowest dose protocol was used for the subsequent analyses.

### 3.3 Motion quantification error versus temporal resolution

**FIGURE 4:**
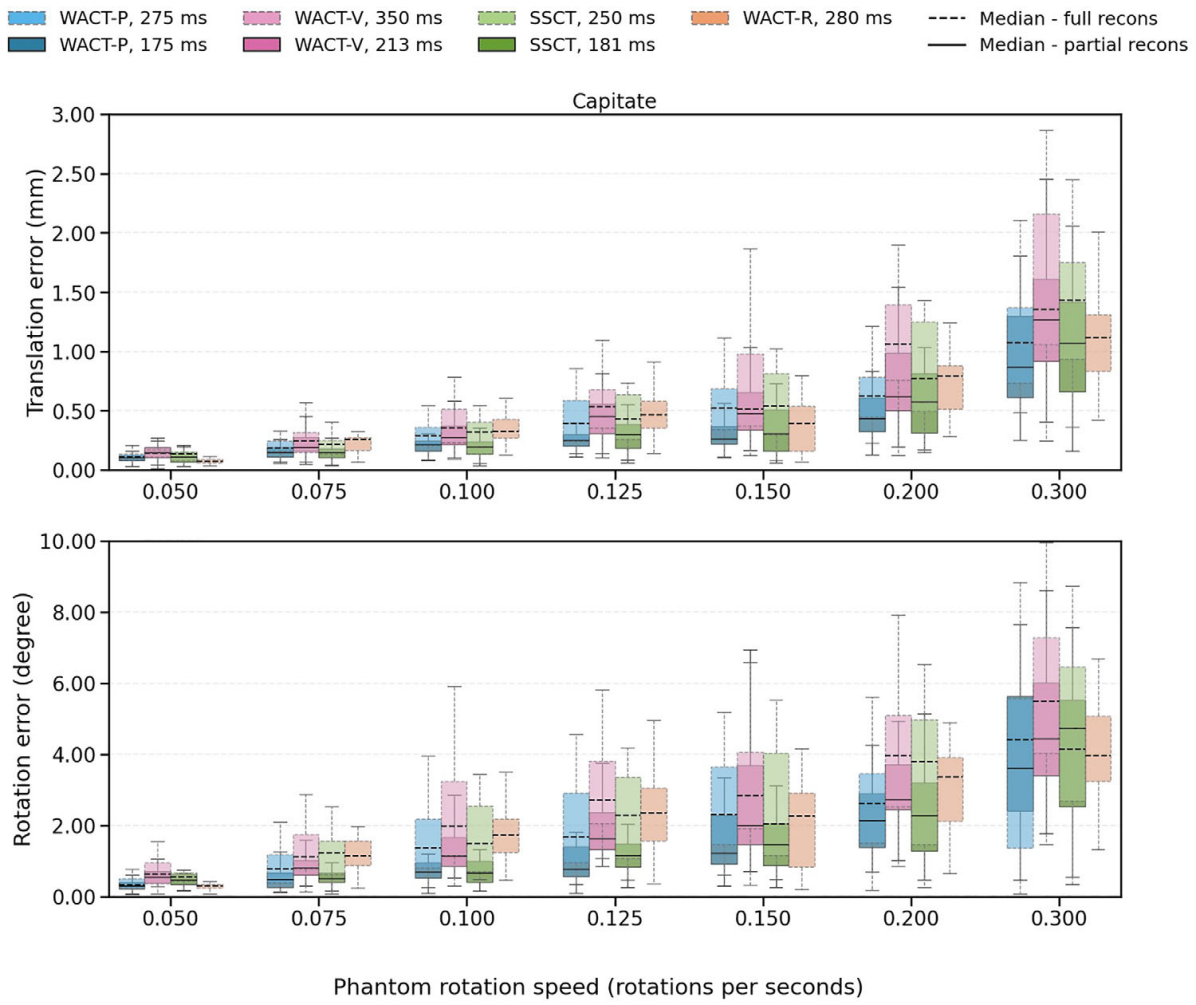
Motion quantification errors following 4D CT acquisition and analysis for the capitate relative to the lunate (reference) in a rotating phantom, using single‐source scanning (WACT‐P, WACT‐V, SSCT, WACT‐R). Boxplots show the distributions for reconstructions from full rotation (dashed) and partial rotation acquisitions (solid). The central line represents the median, the box indicates the interquartile range (Q1–Q3), and the whiskers extend to 1.5× the interquartile range.

Figure 4 shows the translation and rotation errors of the capitate with respect to the lunate (reference) for the SSCT systems (WACT‐P, WACT2, WACT‐R and SSCT). For both translational and rotational errors, phantom rotation speed showed the largest effect (partial η^2^ = 0.69 and 0.79, respectively), followed by bone type (partial η^2^ = 0.56 and 0.58), and reconstruction method (partial η^2^ = 0.06 and 0.17). All three factors reached statistical significance (*p* < 0.05). Capitate showed significantly higher errors than scaphoid for both translation (0.46 mm vs. 0.14 mm, *p* < 0.001) and rotation (1.93° vs. 0.82°, *p* < 0.001). Full reconstructions showed significantly higher errors than partial reconstructions for both translation (0.34 mm vs. 0.27 mm, *p* = 0.027) and rotation (1.60° vs. 1.14°, *p* < 0.001). Across the majority of the single‐source scanners, full reconstructions resulted in higher median errors compared to those from partial reconstruction. The partial reconstruction of WACT‐P and SSCT resulted in the lowest error across phantom rotation speeds. The median and interquartile range values and the corresponding scaphoid results are included in the supplementary material.

**FIGURE 5:**
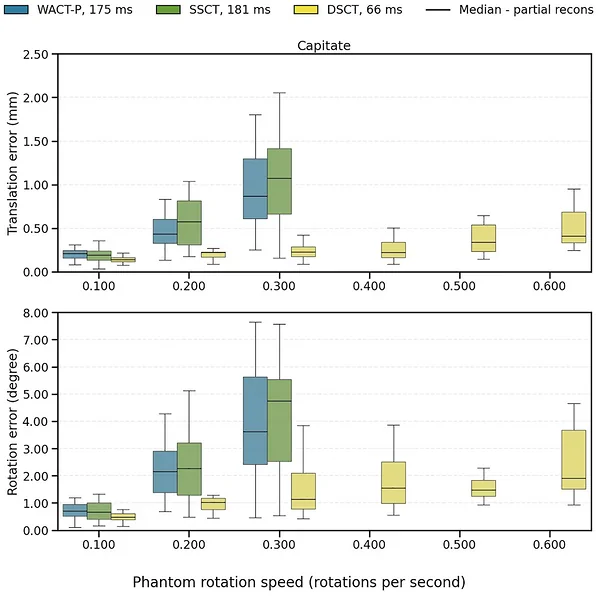
Translation and rotation errors following 4D CT acquisition and analysis for the capitate relative to the lunate (reference) in a rotating phantom showing the two partial‐rotation reconstructions in the single‐source CT system with the lowest error (WACT‐P, SSCT), together with the dual‐source system (DSCT). Boxplots show the distributions for reconstructions from partial‐rotation (solid) reconstructions. The central line represents the median, the box indicates the interquartile range (Q1–Q3), and the whiskers extend to 1.5× the interquartile range.

DSCT showed numerically lower translational and rotational errors compared to the partial reconstructions of WACT‐P and SSCT, although these differences did not reach statistical significance (translation: *p* = 0.12; rotation: *p* = 0.07), (Figure 5).

**FIGURE 6:**
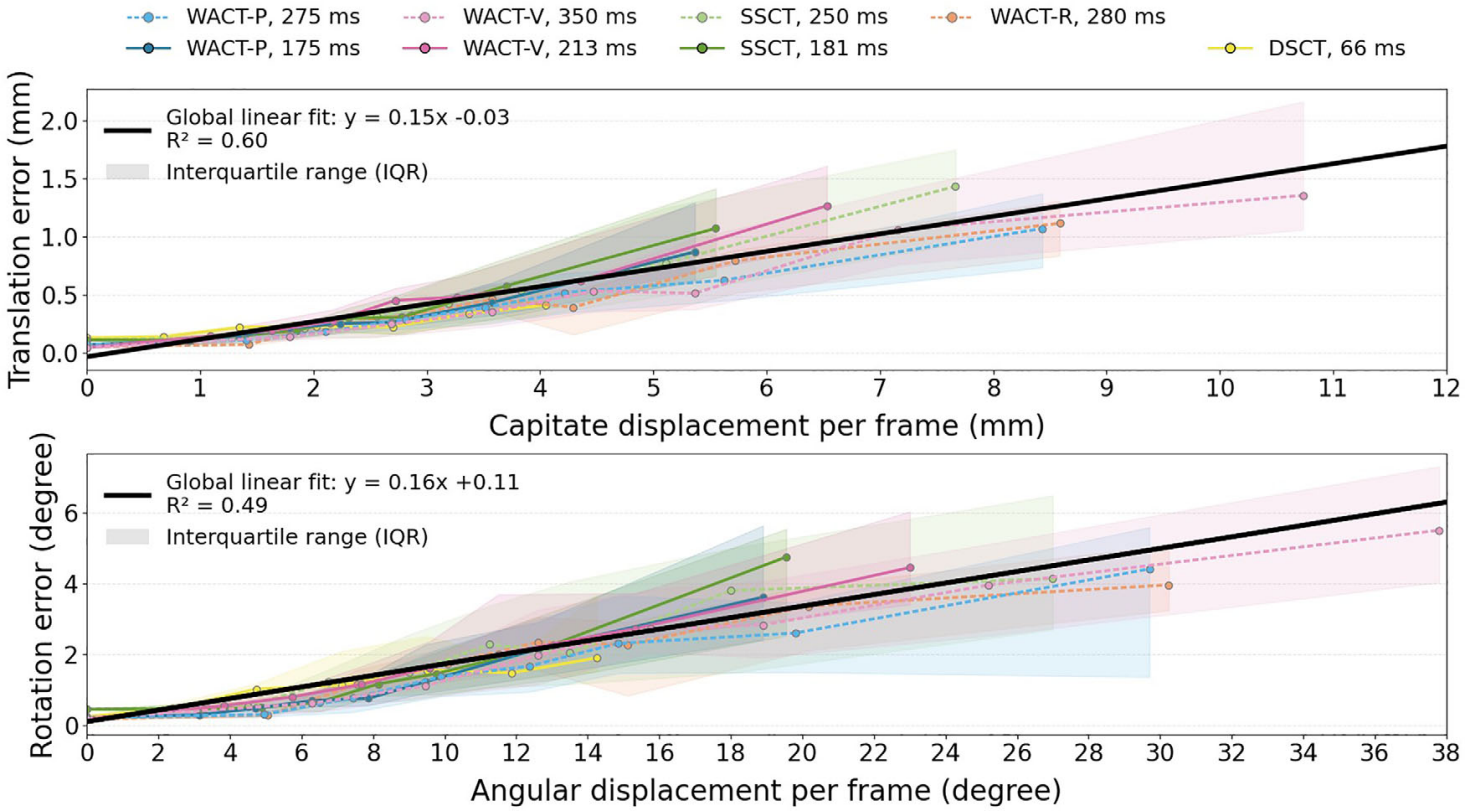
Translation and rotation errors following 4D CT acquisition and analysis for the capitate relative to the lunate (reference) in a rotating phantom, shown as function of capitate displacement in mm (top) and angular displacement in degrees (bottom) per reconstructed frame. Displacement per frame was calculated from the phantom rotation speed and the scanner temporal resolution, using the distance between the rotation axis and the capitate centroid (16.3 mm) for the translation. Lines indicate median translation error per scanner and reconstruction protocol (dashed: full reconstruction; solid: partial reconstruction); shaded regions indicate the interquartile range (Q1–Q3); black regression lines indicate the global linear relationship across all scanners and protocols.

Figure 6 demonstrates the relationship between translation and rotation errors of the capitate, and the normalized motion parameter across the different scanner systems and reconstruction protocols. Translation errors increased consistently with increasing capitate displacement occurring during acquisition of one frame. Pooled linear regression analysis demonstrated a linear relationship, described by E_trans_ = 0.15*x*−0.03 (*R* ^2^ = 0.60), where x represents the capitate displacement per frame in millimeters. Similarly, rotation errors increased with increasing angular displacement per acquisition of one frame, with a linear relationship described by E_rotation_ = 0.16*x* + 0.11 (*R* ^2^ = 0.49).

## 4 DISCUSSION

In this study, we investigated the motion quantification accuracy across acquisition and reconstruction protocols for different CT systems in 4D CT, using a wrist bone phantom. By quantifying motion errors under controlled phantom rotation speeds, we demonstrated that phantom rotation speed, and thus the amount of motion per reconstructed frame, was the dominant factor, while different acquisition protocols did not significantly influence motion quantification accuracy. Furthermore, the relationship between the motion quantification error and motion per frame was largely independent of scanner system.

In dynamic wrist imaging, automated segmentation and registration are important for clinical implementation, as the large data volume and multi‐bone anatomy make manual analysis time‐intensive and difficult to integrate into routine clinical workflows. However, before quantitative wrist kinematics can be interpreted clinically, the effects of motion artifacts on motion quantification accuracy must be understood, as small measured inter‐bone motions may reflect measurement uncertainty rather than true physiological motion. The present study addresses this by quantifying the motion‐dependent error across multiple CT systems and acquisition protocols and by providing scanner‐ and protocol‐specific reference values for motion quantification errors in dynamic wrist CT.

The use of partial reconstruction in case of dynamic wrist imaging has been previously described in several studies, primarily because the reduced reconstruction angle improves temporal resolution and thereby reduces motion‐induced artifacts in reconstructed frames.^11^, ^23^, ^24^, ^28^ In the present study, for almost all phantom rotation speeds in the single‐source systems, the partial‐reconstructed scans (with improved temporal resolution compared to full reconstructions) yielded smaller motion quantification errors. The only exception was one scanner (SSCT) at the highest phantom rotation speed, for which the partial reconstruction showed a slightly higher median error than the full reconstruction. However, the normalized analysis in Figure 6 suggests that improved temporal resolution alone does not necessarily result in lower translation errors. For the same amount of motion occurring within a reconstructed frame, partial reconstructions showed slightly higher errors than full reconstruction. This observation is consistent with known characteristics of partial‐scan reconstruction, where the reduced reconstruction angle improves temporal resolution, but also decreases angular sampling, thereby increasing sensitivity to projection inconsistencies (Figure 2).^23^, ^29^ In static imaging, such effects can manifest as periodic bone positioning errors, as described by Dobbe et al.^22^ In our static experiments, all five CT systems demonstrated a translation error below 0.15 mm and a rotation error below 0.5° (Figure 3). These errors remained substantially smaller than the motion quantification errors observed during dynamic acquisitions and below the kinematic differences reported between healthy and unstable wrists.^30^ Nevertheless, scanner‐specific differences (e.g., gantry stability) may still influence the occurrence of periodic artifacts. Therefore, improved temporal resolution alone should not determine the choice of partial reconstruction, as reconstruction‐related artifacts may offset the benefits. Before partial reconstruction protocols are implemented clinically, static phantom acquisitions should be evaluated for each scanner system.

Moreover, expressing the results in terms of normalized motion parameters allowed motion quantification error to be related to a given amount of motion, independent of scanner‐specific temporal resolution. Translation errors were expressed as a function of capitate displacement per frame, whereas rotation errors were expressed as a function of angular displacement per frame, providing a radius‐independent representation of rotational motion. Despite these different motion measures, the translation and rotation analysis exhibited similar slopes, indicating that the motion quantification error scaled proportionally with the underlying motion, which corresponds to approximately 15% of the motion occurring during one reconstructed frame. Thus, the measured error can be interpreted relative to the amount of motion present.

The optimal wrist rotation speed involves a trade‐off: slower motion reduces motion artifacts and improves motion quantification accuracy, whereas excessively slow motion no longer reflects typical wrist motion during daily activities and may reduce the diagnostic value of the scan. Within wrist speeds currently used in clinical protocols (0.50–1.50 motion cycles per 10 s),^10^, ^12^, ^14^ corresponding to phantom rotation speeds of 0.050–0.150 rotations per second, partial‐reconstructions in the single‐source systems resulted in absolute translation errors between 0.26 mm–0.48 mm. For context, manual 2D measurements of SL distance reported optimal diagnostic cutoffs as 1.65 mm^16^ and mean SL gaps of 2.2–2.5 mm in mild instability versus 3.9 mm in severe instability.^31^ Automated 3D kinematic analyses report smaller differences, with SL distances increasing from approximately 2.3 mm in uninjured wrists to approximately 3.0 mm in injured wrists during extension,^15^ while dynamic MRI demonstrated mean SL distances below 1.8 mm in healthy wrists, compared with 3.3 mm and higher in partial and complete SL ligament ruptures.^7^ Across all these studies, the reported differences between healthy and unstable wrists exceed the motion quantification errors measured in this study, supporting the ability of dynamic wrist CT to detect clinically relevant SL abnormalities.^14^, ^32^, ^33^ However, it is important to keep in mind that the measured motion quantification errors represent a minimum estimate of the measurement uncertainty, which may be higher in clinical settings due to additional anatomical complexity and less controlled motion. Nevertheless, the phantom setup enabled direct benchmarking of scanner systems under identical, known, and reproducible motion conditions, providing a reference for interpreting clinical kinematic measurements and a basis for further work toward clinical implementation of 4D CT wrist imaging.^18^

Motion quantification accuracy also depends on the bone being analyzed. The capitate showed significantly larger translation and rotation errors than the scaphoid. This can be explained by its larger distance from the phantom's axis of rotation (centroid of the lunate), reflecting the geometric dependence of motion artifacts. This finding underlines the need to consider bone position when selecting the wrist rotation speed for a 4D CT scan. Furthermore, although the clinically implemented acquisition protocols differed between scanner systems, radiation dose did not significantly affect the motion quantification error (*p* < 0.76). This suggests that differences in acquisition parameters had only a limited influence on the motion quantification accuracy. Consequently, a low‐dose protocol can be used for the 4D CT acquisition, while the spiral 3D scan requires standard dose to ensure sufficient image quality for bone segmentation. As a result, bilateral 4D CT can be performed with an effective dose of approximately 0.05 mSv–0.10 mSv for single‐source systems.^9^, ^34^

A statistically significant difference between the single‐source and DSCT systems was observed, with DSCT achieving the highest motion quantification accuracy because of its high temporal resolution (66 ms). However, the current DSCT implementation requires ECG triggering, which limits the acquisition time to less than 2 s. To capture a full motion cycle within the acquisition time of the dual‐source protocol, the wrist would need to move at a speed of around 0.500 rotations per second (clinically equal to roughly 5 motion cycles per 10 s). At such rotation speeds, the observed translation error ranged between 0.24 mm and 0.54 mm, which may limit the reliable interpretation of the intercarpal kinematics (Figure 5). Consequently, the short acquisition timeframe limits the clinical advantage of DSCT over single‐source scanners in clinical practice. Additionally, the limited *z*‐axis coverage of the DSCT system (5.52 cm) may complicate acquisitions requiring sufficient radius coverage for reliable automatic registration.^35^ Cardiac acquisition protocols on single‐source scanners for musculoskeletal imaging has previously been explored by Keelson et al.,^24^ demonstrating reduced motion artifacts compared to conventional cine mode. In cardiac CT, motion‐compensation techniques can achieve an effective temporal resolution beyond the gantry rotation time by combing partial projection data with estimated motion vector fields derived from the periodic cardiac cycle.^36^, ^37^ However, such approaches are, for the most part, not directly transferable to non‐cardiac dynamic CT yet, since wrist motion is likely to be non‐periodic and therefore lacks reproducible cycles.

This study has limitations. First, the experiments were performed on a wrist phantom with 3D‐printed bones rather than on cadaveric specimens or in vivo anatomy. Although the contrast between the 3D‐printed bones and surrounding air was comparable to the contrast between cortical bone and soft tissue in clinical wrist CT (i.e., in der order of 1000 HU), the attenuation properties of the individual materials differ. The results should therefore primarily be interpreted as an assessment of motion quantification errors rather than a direct validation of segmentation and registration performance in vivo. Secondly, the experiments were performed under controlled phantom conditions representing a simple, uniform rotational motion. In clinical practice, wrist motion speed may vary within a single scan. However, because segmentation is performed on the static 3D image and each 4DCT frame is registered independently, motion quantification is primarily determined by the amount of motion within each frame and the resulting motion artifacts, rather than by the overall periodicity of the motion pattern. The relationship between motion speed, temporal resolution, and motion quantification error therefore remains relevant in clinical practice, including when wrist motion is irregular or unpredictable. Lastly, scanners from three vendors were included. Broader evaluation across other vendors will be necessary to establish the generalizability of dynamic wrist CT protocols. Nevertheless, by including an older scanner model (WACT‐V), we demonstrated that dynamic wrist imaging is not restricted to the newest models, suggesting that adoption of dynamic imaging might be possible in the majority of hospitals and not only in centers with the most advanced equipment. Future studies could extend this work by using cadaveric arms with an automated motion setup to more closely mimic clinical wrist motion.

## 5 CONCLUSIONS

This study showed that, within the range of conditions studied, motion quantification accuracy in 4D CT imaging of a wrist phantom was primarily determined by the amount of motion occurring per reconstructed frame. Motion quantification error increased approximately linearly with normalized motion per frame, regardless of CT system or reconstruction protocol. Overall, single‐source 4D CT protocols appear to provide sufficient accuracy for quantitative analysis of wrist motion. However, when selecting the wrist rotation speed, it is important to consider the trade‐off between minimizing motion artifacts and preserving physiologically relevant motion. These findings may provide practical guidance for clinical implementation of dynamic wrist CT imaging by defining the motion‐dependent detection threshold that quantitative wrist kinematics should exceed before being interpreted as true physiological motion rather than artifact‐driven displacement.

## CONFLICT OF INTEREST STATEMENT

Ioannis Sechopoulos has research agreements with Siemens Healthcare, Canon Medical Systems, ScreenPoint Medical, Lunit, and DeepHealth, speaker agreements with Siemens Healthcare and Canon Medical Systems, and is a Scientific Advisory Board member of Koning Corp.

## Supporting information




**Supporting Information**: mp70667‐sup‐0001‐Figure SA1.jpg

**Supporting Information**: mp70667‐sup‐0002‐Figure SA2.jpg

**Supporting Information**: mp70667‐sup‐0003‐Figure SC1.jpg

**Supporting Information**: mp70667‐sup‐0004‐Figure SD1.jpg

**Supporting Information**: mp70667‐sup‐0005‐Tables.docx
