# Supplementary material for: Accuracy of motion quantification in dynamic CT images of the wrist joint across multiple CT vendors: A phantom study
Source: Med Phys. 2026 Sep 3;53(9):e70667. doi: 10.1002/mp.70667 (PMC13542767; doi:10.1002/mp.70667)
Supplement: Supplementary file 1 [file MP-53-0-s002.docx]

Supplementary tables:

| **Table SA1**. Dose settings used for 4D CT acquisitions for the WACT-P and DSCT systems | | | | | |
| --- | --- | --- | --- | --- | --- |
| **Scanner** | **Tube voltage [kV]** | **Tube current time product [mAs]** | **Temporal resolution [ms]** | **Acquisition duration [s]** | **CTDI_vol_ [mGy]** |
| WACT – P | 80 | 11 | 275 *(full)*  175 *(partial)* | 10.20 | 4.80 |
|  | 120 | 30 | 275 *(full)*  175 *(partial)* | 10.20 | 57.80 |
| DSCT | 80 | 30 | 66 | 1.65 | 2.90 |
|  | 80 | 75 | 66 | 1.65 | 7.29 |
|  | 80 | 300 | 66 | 1.65 | 29.10 |
|  | 120 | 75 | 66 | 1.65 | 26.45 |
|  | 120 | 300 | 66 | 1.65 | 105.92 |
| **WA** = wide angle; **SS** = single source; **DS** = dual source | | | | | |

| Table SB2A: Translation error of the scaphoid in mm for all CT systems | | | | | | | | |
| --- | --- | --- | --- | --- | --- | --- | --- | --- |
|  | | | | | | | | |
|  | Median [Q1 – Q3] Translation Error (mm) | | | | | | | |
|  | WACT-P | | WACT-V | | WACT-R | SSCT | | DSCT |
| Temporal resolution [ms] | 275 | 175 | 350 | 213 | 280 | 250 | 181 | 66 |
| Phantom rotation speed [rps] |  |  |  |  |  |  |  |  |
| 0.050 | 0.06  [0.05–0.09] | 0.07  [0.06–0.08] | 0.08  [0.05–0.10] | 0.07  [0.05–0.10] | 0.04  [0.03–0.05] | 0.06  [0.05–0.07] | 0.04  [0.04–0.05] | - |
| 0.075 | 0.08  [0.06–0.10] | 0.07  [0.05–0.10] | 0.09  [0.07–0.13] | 0.09  [0.07–0.12] | 0.08  [0.07–0.11] | 0.08  [0.06–0.11] | 0.06  [0.05–0.09] | - |
| 0.100 | 0.10  [0.07–0.12] | 0.10  [0.07–0.13] | 0.12  [0.10–0.15] | 0.10  [0.09–0.15] | 0.11  [0.08–0.13] | 0.12  [0.08–0.20] | 0.08  [0.06–0.10] | 0.12  [0.11–0.13] |
| 0.125 | 0.13  [0.09–0.18] | 0.11  [0.08–0.14] | 0.17  [0.10–0.23] | 0.14  [0.10–0.19] | 0.13  [0.10–0.16] | 0.13  [0.10–0.23] | 0.09  [0.06–0.13] | - |
| 0.150 | 0.16  [0.11–0.18] | 0.16  [0.11–0.22] | 0.16  [0.11–0.20] | 0.20  [0.13–0.25] | 0.12  [0.09–0.14] | 0.15  [0.12–0.26] | 0.10  [0.08–0.15] | - |
| 0.200 | 0.18  [0.12–0.24] | 0.13  [0.10–0.24] | 0.28  [0.17–0.38] | 0.26  [0.19–0.33] | 0.18  [0.12–0.25] | 0.24  [0.19–0.29] | 0.13  [0.09–0.24] | 0.08  [0.06–0.11] |
| 0.300 | 0.26  [0.20–0.34] | 0.33  [0.17–0.45] | 0.50  [0.38–0.94] | 0.46  [0.36–0.56] | 0.20  [0.15–0.33] | 0.38  [0.25–0.52] | 0.22  [0.14–0.35] | 0.11  [0.08–0.13] |
| 0.400 | - | - | - | - | - | - | - | 0.14  [0.11–0.16] |
| 0.500 | - | - | - | - | - | - | - | 0.14  [0.11–0.21] |
| 0.600 | - | - | - | - | - | - | - | 0.18  [0.12–0.19] |

| Table SB2B: Translation error of the capitate in mm for all CT systems | | | | | | | | |
| --- | --- | --- | --- | --- | --- | --- | --- | --- |
|  | Median [Q1 – Q3] Translation Error (mm) | | | | | | | |
|  | WACT-P | | WACT-V | | WACT-R | SSCT | | DSCT |
| Temporal resolution [ms] | 275 | 175 | 350 | 213 | 280 | 250 | 181 | 66 |
| Phantom rotation speed [rps] |  |  |  |  |  |  |  |  |
| 0.050 | 0.11  [0.09–0.13] | 0.10  [0.08–0.12] | 0.14  [0.11–0.17] | 0.15  [0.10–0.19] | 0.07  [0.06–0.09] | 0.13  [0.08–0.15] | 0.11  0.07–0.15] | - |
| 0.075 | 0.18  [0.16–0.24] | 0.15  [0.11–0.19] | 0.25  [0.15–0.32] | 0.19  [0.16–0.28] | 0.26  [0.16–0.28] | 0.21  [0.14–0.25] | 0.15  [0.11–0.18] | - |
| 0.100 | 0.29  [0.21–0.36] | 0.21  [0.16–0.25] | 0.36  [0.23–0.51] | 0.27  [0.22–0.37] | 0.33  [0.27–0.43] | 0.32  [0.13–0.40] | 0.19  [0.14–0.24] | 0.14  [0.12–0.17] |
| 0.125 | 0.39 [0.25–0.58] | 0.25  [0.21–0.30] | 0.53  [0.35–0.68] | 0.45  [0.31–0.56] | 0.46  [0.36–0.58] | 0.43  [0.26–0.64] | 0.30  [0.18–0.39] | - |
| 0.150 | 0.52  [0.34–0.69] | 0.26  [0.22–0.37] | 0.51  [0.37–0.98] | 0.48  [0.34–0.65] | 0.39  [0.16–0.54] | 0.54  [0.30–0.81] | 0.31  [0.16–0.51] | - |
| 0.200 | 0.63  [0.45–0.78] | 0.43  [0.33–0.60] | 1.06  [0.76–1.40] | 0.62  [0.50–0.99] | 0.79  [0.52–0.88] | 0.77  [0.50–1.25] | 0.58  [0.31–0.82] | 0.22  [0.17–0.23] |
| 0.300 | 1.07  [0.73–1.37] | 0.87  [0.61–1.30] | 1.36  [1.06–2.16] | 1.27  [0.92–1.61] | 1.12  [0.84–1.31] | 1.43  [0.93–1.75] | 1.07  [0.66–1.42] | 0.23  [0.18–0.29] |
| 0.400 | - | - | - | - |  | - | - | 0.22  [0.17–0.34] |
| 0.500 | - | - | - | - |  | - | - | 0.34  [0.24–0.54] |
| 0.600 | - | - | - | - |  | - | - | 0.41  [0.33–0.69] |
| WA = wide angle; SS = single source; DS = dual source | | | | | | | | |

| Table SB3a: Rotation error of the scaphoid in degrees for all CT systems | | | | | | | | |
| --- | --- | --- | --- | --- | --- | --- | --- | --- |
|  | Median [Q1 – Q3] Rotation Error (degrees) | | | | | | | |
|  | WACT-P | | WACT-V | | WACT-R | SSCT | | DSCT |
| Temporal resolution [ms] | 275 | 175 | 350 | 213 | 280 | 250 | 181 | 66 |
| Phantom rotation speed [rps] |  |  |  |  |  |  |  |  |
| 0.050 | 0.26  [0.17–0.33] | 0.26  [0.19–0.31] | 0.36  [0.20–0.42] | 0.29  [0.21–0.41] | 0.28  [0.21–0.35] | 0.31  [0.28–0.42] | 0.34  [0.30–0.39] | - |
| 0.075 | 0.36  [0.26–0.50] | 0.30  [0.24–0.43] | 0.42  [0.32–0.57] | 0.40  [0.23–0.51] | 0.44  [0.34–0.52] | 0.38  [0.31–0.43] | 0.37  [0.31–0.45] | - |
| 0.100 | 0.52  [0.38–0.66] | 0.46  [0.34–0.57] | 0.63  [0.46–0.77] | 0.50  [0.34–0.65] | 0.36  [0.20–0.65] | 0.44  [0.34–0.48] | 0.47  [0.34–0.56] | 0.38  [0.23–0.41] |
| 0.125 | 0.65  [0.47–0.82] | 0.47  [0.35–0.68] | 0.86  [0.58–1.74] | 0.59  [0.37–0.95] | 0.87  [0.56–1.12] | 0.47  [0.38–0.67] | 0.56  [0.34–0.71] | - |
| 0.150 | 0.78  [0.55–1.01] | 0.59  [0.44–0.73] | 1.21  [0.70–1.87] | 0.68  [0.45–1.02] | 0.71  [0.62–0.82] | 0.64  [0.45–0.92] | 0.75  [0.57–0.89] | - |
| 0.200 | 1.00  [0.80–1.80] | 0.95  [0.68–1.35] | 1.84  [1.05–3.45] | 1.13  [0.77–1.68] | 1.30  [0.58–1.96] | 1.44  [0.86–2.32] | 1.00  [0.46–1.14] | 0.41  [0.30–0.52] |
| 0.300 | 1.69  [1.29–3.23] | 1.33  [0.88–3.25] | 4.43  [2.32–7.00] | 2.56  [1.62–3.78] | 1.32  [0.86–2.54] | 2.76  [1.78–4.37] | 1.53  [1.09–2.05] | 0.69  [0.49–0.75] |
| 0.400 | - | - | - | - | - | - | - | 0.74  [0.53–0.83] |
| 0.500 | - | - | - | - | - | - | - | 0.80  [0.56–0.83] |
| 0.600 | - | - | - | - | - | - | - | 1.35  [1.06–2.07] |
| WA = wide angle; SS = single source; DS = dual source | | | | | | | | |

| Table SB3B: Rotation error of the capitate in degrees for all CT systems | | | | | | | | |
| --- | --- | --- | --- | --- | --- | --- | --- | --- |
|  | Median [Q1 – Q3] Rotation Error (degrees) | | | | | | | |
|  | WACT-P | | WACT-V | | WACT-R | SSCT | | DSCT |
| Temporal resolution [ms] | 275 | 175 | 350 | 213 | 280 | 250 | 181 | 66 |
| Phantom rotation speed [rps] |  |  |  |  |  |  |  |  |
| 0.050 | 0.32  [0.25–0.51] | 0.31  [0.23–0.40] | 0.63  [0.38–0.95] | 0.56  [0.40–0.72] | 0.30  [0.24–0.35] | 0.55  [0.45–0.67] | 0.47  [0.35–0.63] | - |
| 0.075 | 0.78  [0.38–1.18] | 0.49  [0.26–0.67] | 1.11  [0.80–1.75] | 0.81  [0.61–1.02] | 1.15  [0.88–1.56] | 1.22  [0.64–1.57] | 0.51  [0.41–0.68] | - |
| 0.100 | 1.37  [0.82–2.18] | 0.70  [0.53–0.95] | 1.98  [1.16–3.25] | 1.15  [0.85–1.66] | 1.74  [1.24–2.19] | 1.49  [0.71–2.56] | 0.66  [0.41–1.00] | 0.48  [0.39–0.62] |
| 0.125 | 1.67  [0.95–2.93] | 0.77  [0.57–1.42] | 2.72  [2.07–3.82] | 1.63  [1.32–2.37] | 2.35  [1.56–3.06] | 2.28  [1.07–3.37] | 1.17  [0.83–1.49] | - |
| 0.150 | 2.31  [1.48–3.65] | 1.23  [0.91–2.28] | 2.83  [1.91–4.06] | 2.01  [1.48–3.69] | 2.27  [0.84–2.91] | 2.05  [1.16–4.05] | 1.46  [0.88–2.05] | - |
| 0.200 | 2.60  [1.50–3.46] | 2.15  [1.39–2.90] | 3.95  [2.53–5.10] | 2.73  [2.45–3.72] | 3.36  [2.11–3.92] | 3.79  [1.48–4.99] | 2.28  [1.29–3.21] | 1.03  [0.76–1.19] |
| 0.300 | 4.41  [1.37–5.58] | 3.61  [2.42–5.63] | 5.50  [4.03–7.29] | 4.45  [3.41–6.02] | 3.96  [3.24–5.07] | 4.14  [2.70–6.47] | 4.74  [2.53–5.53] | 1.14  [0.79–2.09] |
| 0.400 | - | - | - | - | - | - | - | 1.56  [0.99–2.52] |
| 0.500 | - | - | - | - | - | - | - | 1.48  [1.26–1.83] |
| 0.600 | - | - | - | - | - | - | - | 1.91  [1.52–3.67] |
| WA = wide angle; SS = single source; DS = dual source | | | | | | | | |
